# Tectonic evolution and paleokarstification of carbonate rocks in the Paleozoic Tarim Basin

**DOI:** 10.1007/s13146-016-0307-4

**Published:** 2016-07-14

**Authors:** Xuhui Xu, Qianglu Chen, Chenglin Chu, Guorong Li, Cunge Liu, Zheng Shi

**Affiliations:** 1 Wuxi Research Institute of Petroleum Geology, SINOPEC, Wuxi, 214126 Jiangsu China; 2 Energy Resources of Chengdu University of Technology, Chengdu, 610059 Sichuan China; 3 Exploration and Development Research Institute of NWBC, SINOPEC, Urumqi, 830011 Xinjiang China

**Keywords:** Carbonate rock paleokarst, Ordovician, Tectonic evolution, Paleozoic, The Tarim Basin

## Abstract

Thick carbonate rocks were developed in the depression of the Tarim craton during the Cambrian–Middle Ordovician periods. The compressional tectonic movement during the Middle Caledonian–Hercynian created the paleouplifts, which became the base for the paleokarst in the Ordovician carbonate rocks. Based on the large quantity of seismic, drilling, and geological outcrop data, this study analyzed the paleokarst development in relation to the multi-stage tectonic movements in the Paleozoic Era and different stages of karstification and hypothesized paleogeomorphology and paleokarst water system of those stages. Fractures from the tectonic movements in the carbonate and non-carbonate rocks were essential for water cycle, and therefore, the karst development in deep carbonate rocks. Paleokarsts in the Tarim Basin can be classified into four major types based on the paleogeomorphology, degree of karstification, and the layering, i.e., Tahe type, gentle hill type, high steep hill type, and covered-semi-open type. Relatively, the Tahe type was mostly on hill slopes and had the strongest karstification, the gentle hill type often located in the plain areas or basin bottoms and had least karstification, the high steep hill type was controlled by faults and had medium karstification, the semi-open type was controlled by precipitation and hydraulic gradient, and fracture passages and karst caves were mostly developed along major fractures. Overall, the paleokarsts of the Ordovician carbonate rocks in the Tarim Basin can be characterized by long geologic history, multiple development stages, deep burial depth, and various karst types.

## Introduction

Paleokarstification is referred as karstification occurred before Cenozoic to carbonate rocks that were exposed or close to land surface as a result of regional tectonic uplift. It became paleokarst when the karsitified carbonate rocks were later covered by other sedimentary layers due to crustal subsidence (Zhang et al. 2010). Paleokarst is often directly related to the storage and entrapment of hydrocarbon and groundwater, thus becomes the study focus for petroleum geologists and hydrogeologists. Paleokarst, as a petroleum reservoir related with regional unconformity, was broadly discussed at the International Symposium for “Features and Significance of Paleokarst System and Unconformity Surface” held in 1985 (James and Choquette 1988). Chinese scholars studied the characteristics of paleokarst reservoirs and their control for old storages in the Ordos Basin and Tarim Basin in the 1990s (Zhang et al. 1993; Chen et al. 1994; Guo^1996^). Extensive studies have been carried out on paleokarst reservoirs, especially on the development feature of oil reservoirs, the distribution patterns, factors to control the old storage, and methods to predict the oil storage, in the Tarim Basin in the recent decade, along with the progress in oil and gas exploration in the Tahe Oilfield. The Ordovician paleokarst was characterized by old formation time, multiple formation phases, deep buried depth, and high heterogeneity (Chen et al. 2002, 2012; Lin et al. 2002; Yan et al. 2005; Zhang et al. 2007, 2012; Lv et al. 2009; Zhu et al. 2009; Zhou et al. 2009; Qi et al. 2010; He et al. 2010; Ni et al. 2011; Ji et at 2012; Ma et al. 2013; Yang et al. 2014).

The Tarim Basin is located in Xinjiang Province of western China and is the largest oil and gas-bearing sedimentary basin in China, with an area of 5.6 × 10^5^ km^2^. The Tarim Basin was developed on the Pre-Nanhua continent by a series of complex geological movements during the geological period from Nanhua System to Neogene (Xu et al. 2002, 2004; He et al. 2011; Li et al. 2012). A series of regional unconformity surfaces and paleouplift belts were formed during the Paleozoic multi-phase tectonic evolution. Paleokarstification took place in the Cambrian-to-Ordovician carbonate rocks. This study intends to characterize the correlation between the Paleozoic multi-phased tectonic evolution and paleokarstification, which would helpful for better understanding and characterization of the paleokarst development and possible oil reservoirs in the deeply buried carbonate rocks.

## Evolution of the Paleozoic prototype sedimentary basin

**Fig. 1:**
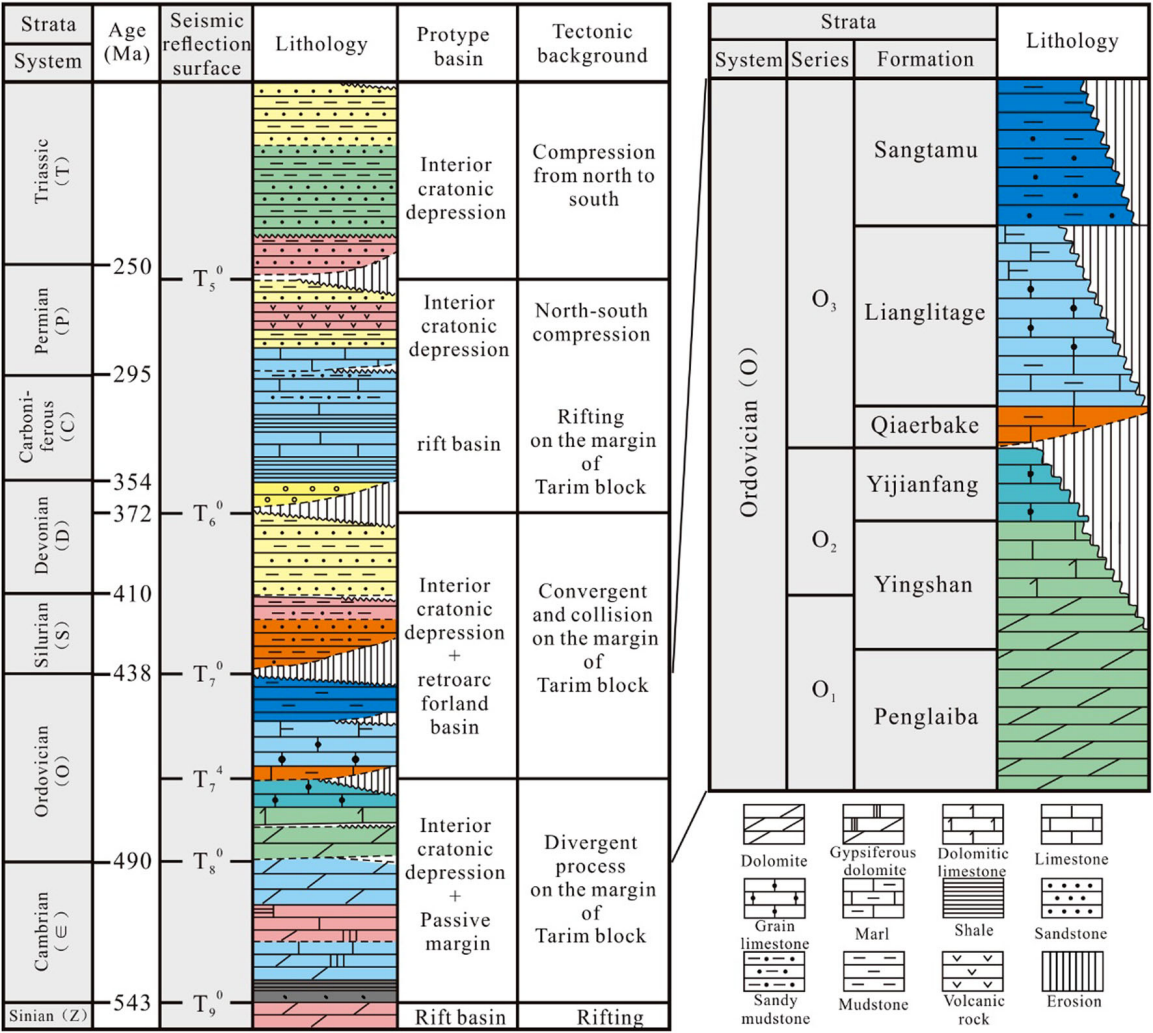
Stratigraphy and lithology of the Paleozoic Sediments in the Tarim Basin

The Tarim Basin is a large superimposed and composite basin that is consisted of the Nanhua–Devonian marine, the Carboniferous-Permian marine-continental transitional, and the Triassic-Quaternary continental sedimentary sequences (Fig. 1). The interior cratonic depression basin and cratonic marginal basin were developed during the Cambrian–Middle Ordovician geological period (Xu et al. 2002; He et al. 2007). The interior cratonic depression basin is composed of thick dolomite, gypseous dolomite, limy dolomite, and limestone in the central-west Tarim Basin. The cratonic marginal basin is consist of siliceous mudstone, limy mudstone, argillaceous limestone, and black shale in the northeast part of the Tarim Basin. There were significant changes of sedimentary facies from late Middle Ordovician to early late Ordovician. The platform facies in the western Tarim Basin was transited into alternating platform—shelf pattern, with Tabei, Tazhong, and Tangnan platform separated by shelves developed from north to south. A large-scale transgression then occurred in the late Ordovician, which flooded the carbonate platforms and formed a very thick deep water shelf and slope facies dark mudstone and calcareous mudstone. The sedimentary facies in the northeast part of the cratonic margin basin were deep water shelf and bathyal system with a large scale of flysch buildups.

The interior cratonic depression and periphery or backarc foreland depression were developed primarily during the Silurian–Middle Devonian period, during which a set of marine clastic rocks, such as shore-shallow marine sandstone, siltstone, mudstone and silty mudstone, and the Carboniferous-Lower Permian interbedded sandstone, mudstone and carbonate rocks, as well as Middle-Upper Permian variegated clastic rocks and multi-layer basalt were developed (Lin et al. 2008; He et al. 2011).

With the goal for understanding the characteristics of karstification during the multi-phased tectonic uplifting process, the analysis in this study primarily focuses on the Ordovician carbonate rocks. The Ordovician strata in the central-western basin can be lithostratigraphically divided into three series and six formations, i.e., the Lower Penglaiba Formation (O_1_p), Middle-Lower Yingshan Formation (O_1_-_2_ys), Middle Yijianfang Formation (O_2_yj), Upper Qiaerbake Formation (O_3_q), Lianglitage Formation (O_3_l), and Sangtamu Formation (O_3_s) (Fig. 1). The Penglaiba Formation is pre-dominantly composed of dolomite. The Yingshan Formation is formed by thick dolomite, limy dolomite, and dolomitic limestone in the lower layer, but thick limestone in the upper layer. The Yijianfang Formation is mainly composed of grainstone, micrite, as well as sponge reef limestone. The Qiaerbake Formation is about 20–50-meters thick and is composed of purple, brownish gray pimple-like argillaceous limestone and argillaceous limestone. The Lianglitage Formation is mainly composed of thick dark gray interbedded calcareous mudstone, thin marl, and greenish gray siltstone-fine sandstone, representing a mixed continental shelf phase.

## Data and research methods

**Fig. 2:**
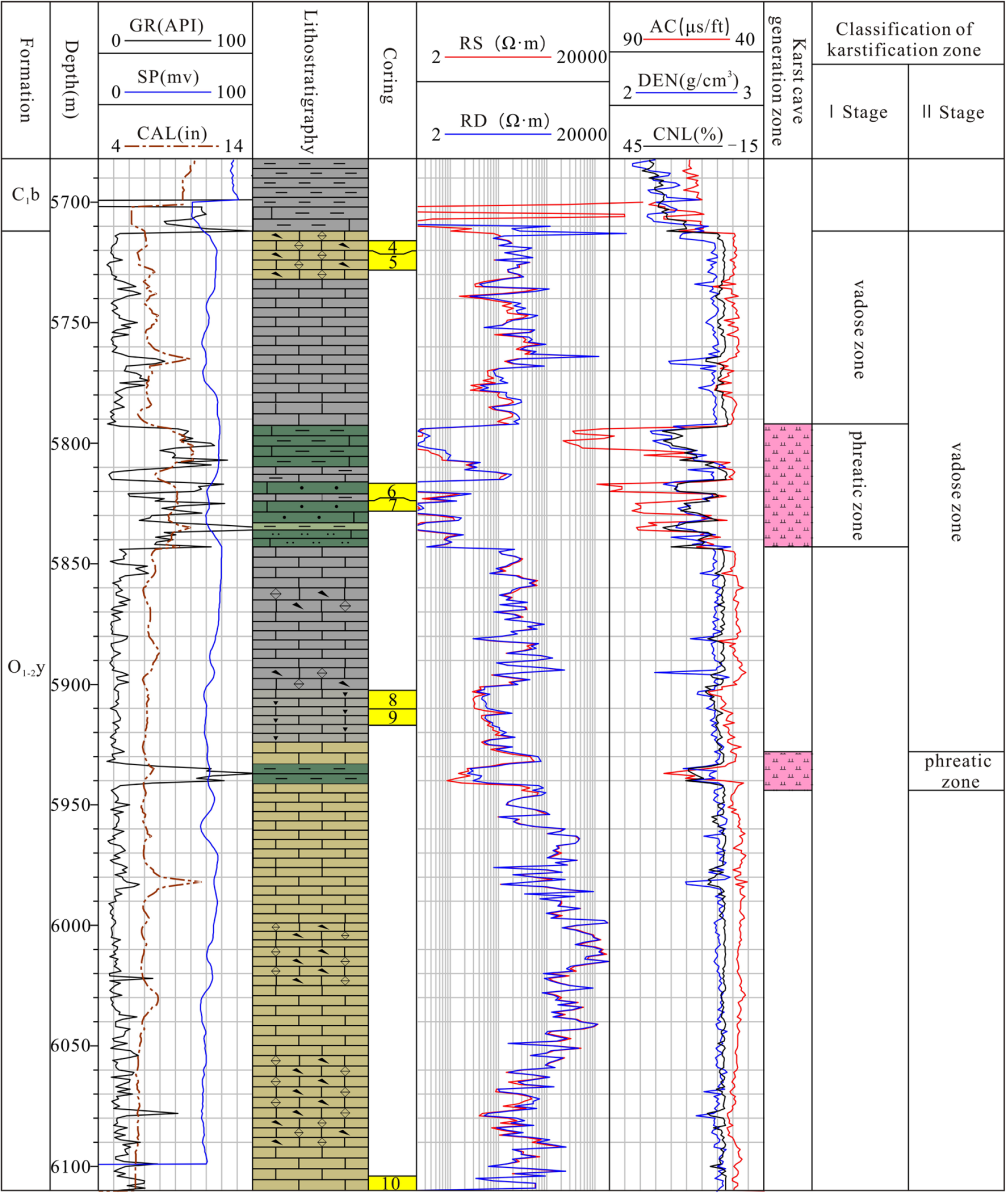
Characteristics of well-loggings as indication of paleokarsts (adopted from Chen et al. 2012)

A large quantity of seismic acquisition and well drillings were conducted in the Tarim Basin for oil and gas exploration in recent years, which made the understanding of the buried paleokarst possible. Analyses on the paleogeomorphology, paleofault, and paleowater system were based on the regional geological setting combined with lithological and paleontological data, and a large quantity of 2D and 3D seismic data (Yan et al. 2005; Zhang et al. 2007, 2012; Lv et al. 2009; Zhu et al. 2009; Qi et al. 2010; He et al. 2010; Ni et al. 2011; Chen et al. 2012; Ji et at 2012; Ma et al. 2013). Carbonate rocks of the Lower Paleozoic are generally buried at a depth greater than 5000 m. Deep drilling well, wire-logging, and seismic data often show signs of paleokarst in these rocks. Signs of paleokarst include loss of drilling bits and drilling fluid (for example, Well AD2 lost 2285 m^3^ of drilling fluid), flooding of drilling well, speed up of drilling time, and low recovery rate of drilling cores. It can be seen from Fig. 2 (Chen et al. 2012) that the CAL and GR values are much higher and the RS value and density were low at the sections, where karstification features presents. Core samples showed voids filled by sandstone and mudstone, karst breccia, chemical sediments (giant crystal calcite, stalactites), weathered fissure, and argillaceous fillings. The discontinuity in seismic profiles showed strong karst cave features, particularly near the top of Ordovician limestone.

## Paleozoic tectonic evolution and paleokarst development

### Regional unconformity and phases of karstification

**Fig. 3:**
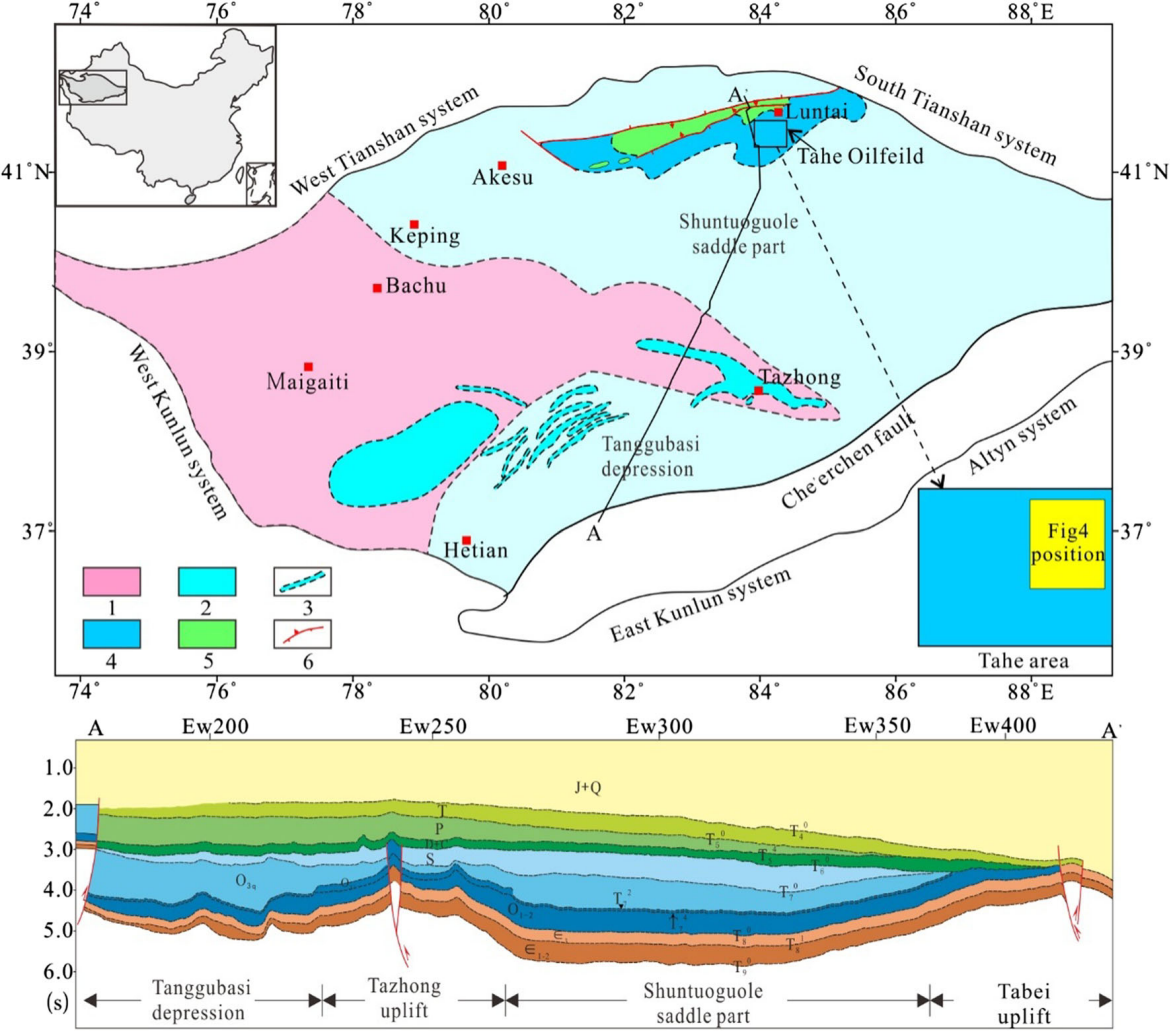
Distribution of paleouplifts (multi-phased angular unconformity) in the Tarim Basin. *1* Uplift formed in the Early Middle Ordovician. *2* Uplift formed in the end of Ordovician. *3* Thrust belt formed in the end of Ordovician and Silurian-Middle Devonian. *4* Uplift formed in the end of Middle Devonian. *5* Uplift formed in the end of Permian

Five major uplifts were formed by tectonic evolutions, respectively, in the later stage of Early and Middle Ordovician, the late Ordovician, the late Middle Devonian, and the late Permian (He et al. 2008, 2011; Lin et al. 2011, 2012, 2013), which lead to complex and diverse stratigraphic features (Fig. 3). Although there are debates on the geologic time for the uplifts in the southwest, center, and north Tarim Basin (Xu et al. 2005; He et al. 2008; Lin et al. 2011), views on the four major unconformities (T_7_^4^, T_7_^0^, T_6_^0^, T_5_^0^) are consistent (Xu et al. 2005; He et al. 2008, 2011; Lin et al. 2011, 2012, 2013). The distribution and intensity of karstification were largely controlled by the unconformity surfaces. Listed in Table 1 are the four distinctive major unconformity surfaces in the Tarim Basin and the corresponding karstification characteristics.

**Table 1:** Cycles of karst development in the Ordovician carbonate rocks of the Tarim Basin

Tectonic movement and regional unconformity surface	Stratigraphic contact (Karst time)	Karstification horizons	Karst development	The main effect scope
end of middle Ordovician/T_7_^4^	O_1–2_ys/O_3_l	O_1–2_ys	Relatively Weak	The South of the Tarim Basin (Markit–Bachu–Tazhong Area)
	O_2_yj/O_3_l	O_2_yj	Weak	Local of Tabei uplift
end of Ordovician/T_7_^0^	O_3_l/S	O_3_l	Moderate	The buried hill belt of NW trending in Tazhong; Markit South
	O_1–2_ys/S	O_1–2_ys	Relatively intense	The buried hill belt of NW trending in Tazhong; Tazhong East
	O_2_yj/S	O_2_yj	Weak	Local of Tabei uplift
end of middle Devonian/T_6_^0^	O_2_yj- O_1–2_ys/C_1_	Upper O_2_yj-O_1–2_ys	Intense	The main part of Tabei Uplift
	O_1–2_ys/C_1_	O_1–2_ys-O_1_p	Intense	The buried hill belt of NW and NE trending in Tazhong; the buried hill belt of NNE-NE trending of Markit
	O_3_l/C_1_	O_3_l	Relatively intense	The buried hill belt of NW and NE trending in Tazhong; (local)
end of Permian/T_5_^0^	O_1_-Cam/T/J/K	O_1_-Cam	Relatively intense	The local area of Tabei Uplift North

### Tectonic evolution, paleogeomorphologic and water systems

Regional tectonic movement could influence the shape of uplift and the scale and strike of faults. At the same time, the karst geomorphology, such as karst forest, karst hills, and valley, formed due to weathering, and erosion could directly influence the hydrogeological condition that controls the development of karst.

Affected by the regional compression in the Earl and Middle Ordovician, the Tarim craton Basin became a structural rift-depression basin, forming a more regional rift and karst plain in the south Tarim Basin. Karstification was mild due to localized faults and lack of dominant fractures. Analysis of mineral data shows that this region was under dry climate condition without abundant surface flow.

The tectonic movement of late Ordovician led to a series of NW and NE belt-shaped thrust folds in Tazhong, such as Tazhong No. II fold. The uplift around the thrust fold could be as much as several kilometers with steep land-surface slopes (Xu et al. 2005; Lin et al. 2011). Karstification mainly took place in fault-fold belt due to the limited development of karst slope and small recharge area of karst water.

**Fig. 4:**
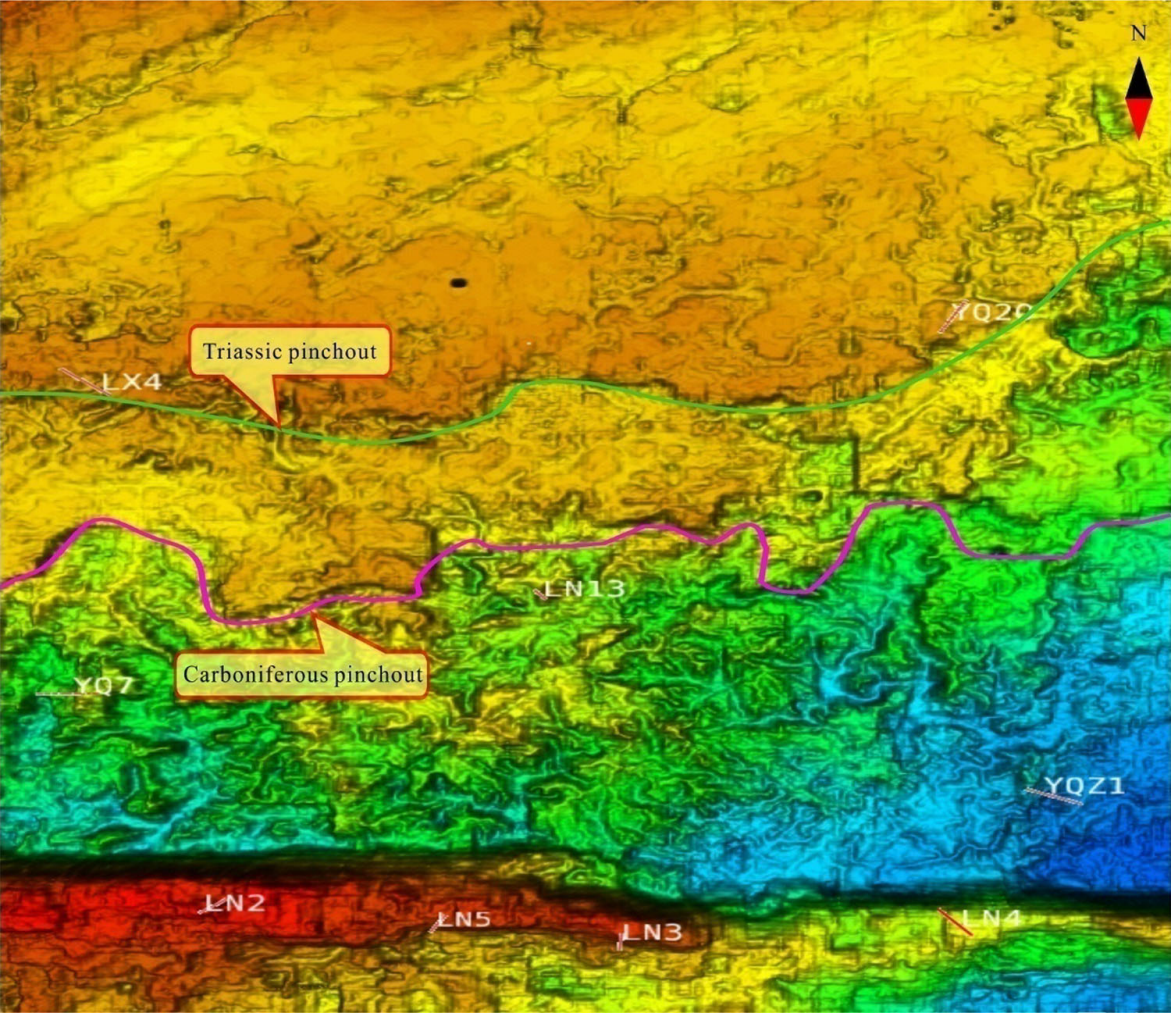
Multi-phase structure and karst geomorphology of the Tabei area

The tectonic compression in late Middle Devonian resulted in a significant uplift in the Tabei area. Large-scale karst landforms, such as karst plateau, karst slope, and karst basin were developed from north to south. Faults and folds developed at the same time intensified weathering of karst landforms to form remnant hills. Moreover, surface water and subsurface karst conduit systems interacted with each other as a couple flow system. More uplifting happened in the Tabei area in the late Permian. The overlying Carboniferous-Permian strata on the Middle Devonian karst plateau in the north of karst plateau were eroded. The Ordovician carbonate rocks were eroded again, which resulted in the destruction of karsts, such as collapse and filling of karst caves. However, the Middle Devonian karst landforms, such as remnant hills and karst forests, were well preserved in the south basin (Fig. 4).

### Control of faults on karst development

The permeability of carbonate rocks along fault zones was higher as a result of fractures created by faulting, karstification occurred near land surface and in the upper infiltration zone, and karst caves were also formed in the deep lateral flow layer by precipitation recharged into the deep layer through faults and fractures (Zhu et al. 2009; Chen et al. 2012; Ji et al. 2012; Dou 2014; Lu et al. 2014). Drilling data into the Ordovician carbonate rocks in the Tabei Rift indicated significant karst features, for example, 91 % of the drilling hit caves (Qi et al. 2010), whereas for buried carbonate rocks, fractures had to exist for water cycle in the system (Lv et al. 2009). For example, in the Tabei Rift, karstification in the middle Devonian was characterized by karst plateau in the north, karst hills in the central region, and karst valley in the south. Karst valley in the south was mostly covered by the O_3_s mudstone, and Silurian–Devonian sandstone and mudstone. Faults, fractures, and elevation difference from north to south created a favorable flow condition for karstification in the covered to semi-open karst system. Drilling data indicated that karstification and karst caves appeared more along the fault zone and were much less away from the fault zone, which implied that faults and fractures were the controlling factors for karstification in the basin (Lv et al. 2009; Qi et al. 2010).

### Types and features of buried paleokarstification

Karstification is controlled by a combination of factors, including the chemical composition of carbonate rocks, formation and types of carbonate rocks, geological structures, hydrologic and climatic conditions. Karst landforms, however, was mostly controlled by the regional geologic structure. As mentioned earlier, the karstification in the Ordovician carbonate rocks can be classified into four major types, i.e., Tahe type (large angular unconformity), gentle hill type (small angular unconformity—paraconformity), Steep-slope hill type (angular unconformity), and covered and semi-open type (buried middle-to-late Ordovician carbonate rocks).

### Tahe type karstification

**Fig. 5:**
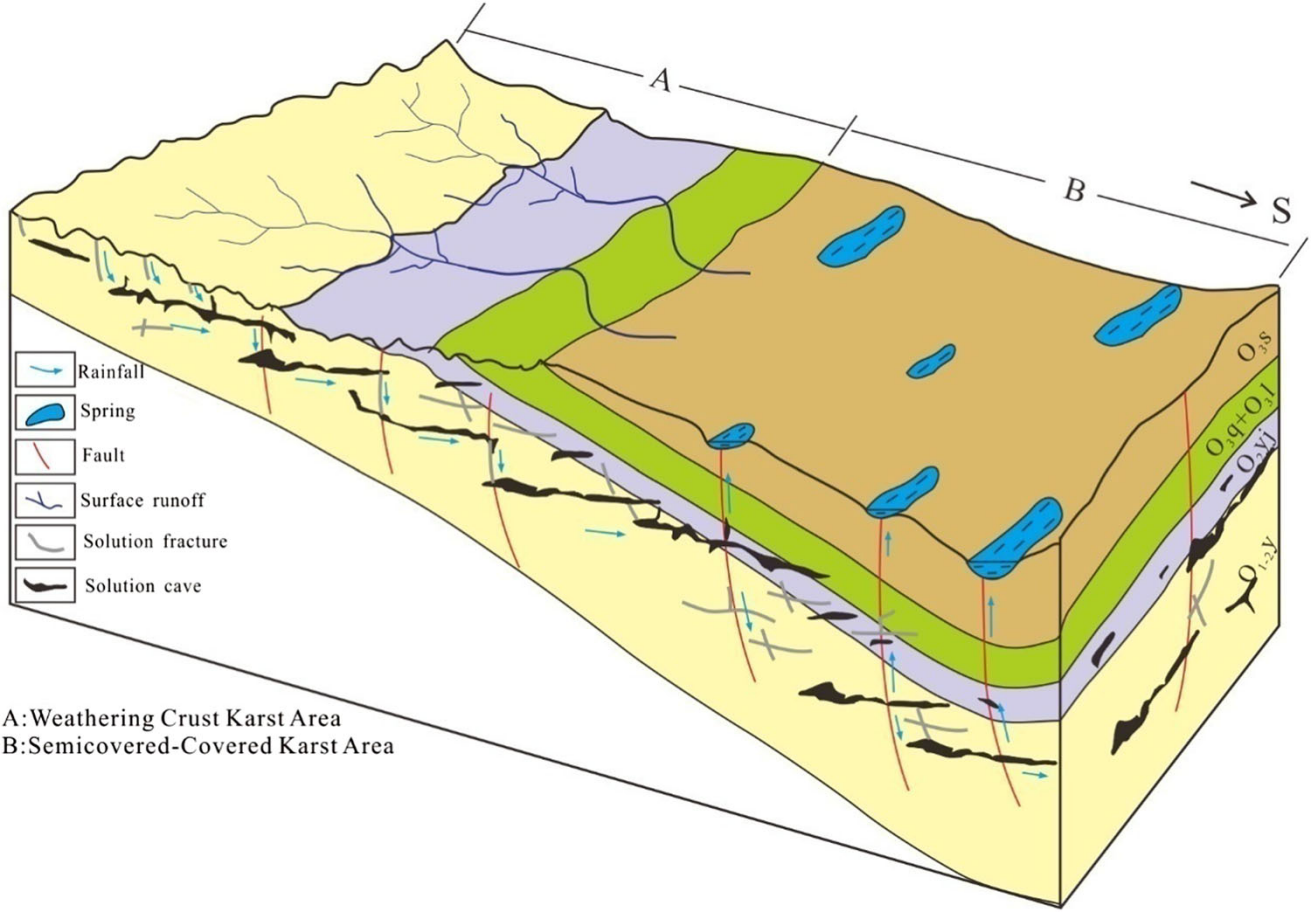
Illustration of Karstification types in the Tarim Basin (Zone A: Tahe type karstification, Zone B: covered and semi-open type karstification)

The Tahe type was located mostly in the Tabei platform. Landforms changed from karst plateau, to karst slope area, and to karst basin, with karst slope being the dominant landform. Karstification was in the well-compacted-thick O_2_yj and O_1–2_ys limestone and the karst stage was around late D_2_ (Table 1). Karsts of this type were featured by: (1) large cave systems. The largest caves without filled sediments interpreted from drilling data were as large as 29.49 meters and 37 meters. The largest filled cave interpreted from drilling core was as large as 20 meters, and interpreted from drilling tests was up to 73 m (Qi et al. 2010). (2) karstification is highly correlated with the unconformity surface. Karsts were mostly developed within the 250-m depth of the unconformity surface. (3) multi-layers of caves. Karsts were controlled by structural movements and cycle of karst developments at various stages. Earlier karst development was during a structurally stable period after intensive uplifting and erosion in late D_2._ Karst features were formed from the weathering zone, to the surface karst zone, phreatic karst zone, and deep slow flow zone. When the karst layer was uplifted, later stage karst development continued on the earlier karst, and the slow flow zone was raised to become the phreatic karst zone, and at the same time, the new slow flow karst zone was developed (Chen et al. 2012) (Fig. 2). 2–3 cycles of karst development could be identified in the Tahe region. Karst features showed high heterogeneity both vertically and horizontally. (4) Karst caves were well preserved. Drilling data showed that 44.2 % of the caves were not filled, only 29.2 % of the caves were fully filled (Qi et al. 2010). Drilling data also showed that filled materials were mostly uncompact, which implied that there was no large cave collapse. Figure 5 illustrated the cycle of karst development in the Tabei region.

### Gentle hill type karstification

**Fig. 6:**
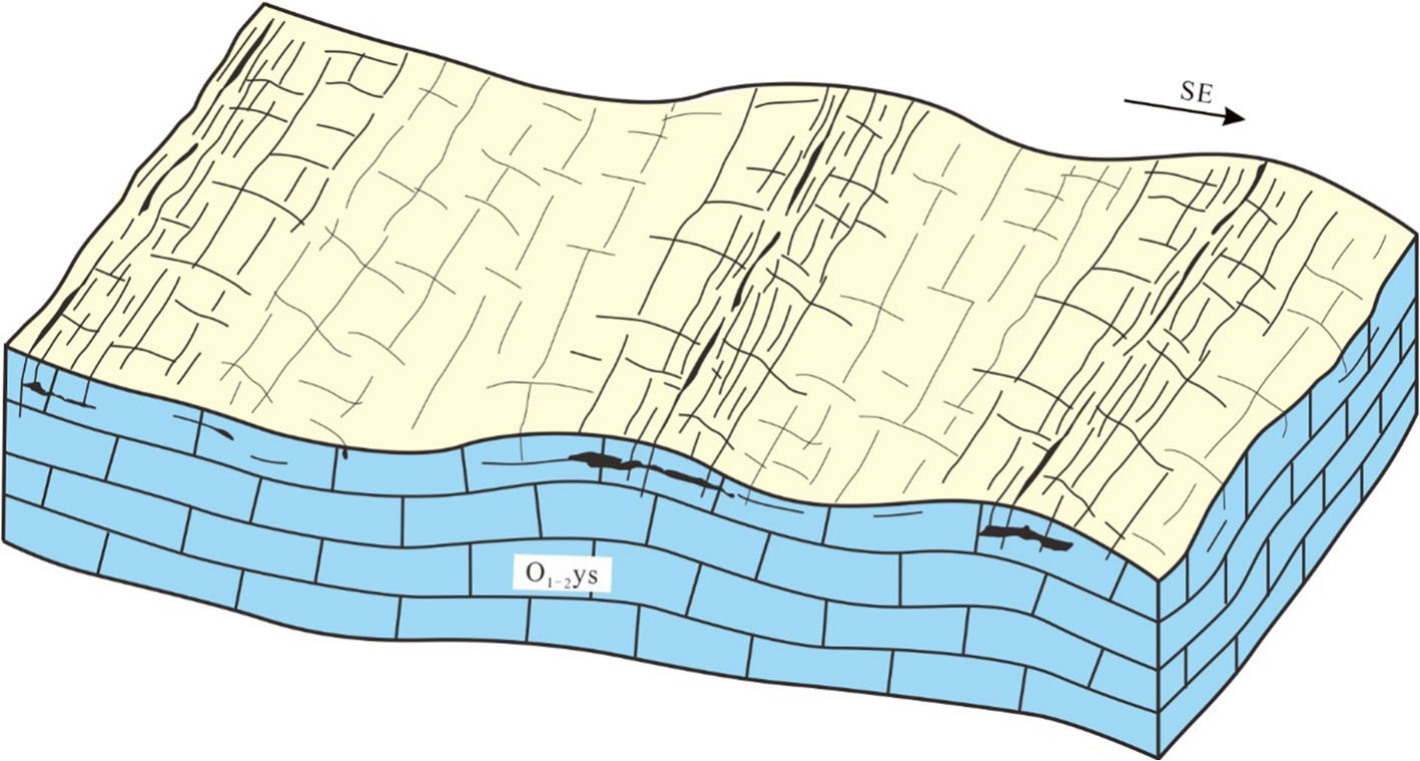
Illustration of gentle hill type karstification

The Tazhong type karsts were widely distributed in the Markit–Bachu–Tazhong area. Karstification occurred pre-dominantly in limestone, dolomitic limestone, and dolomite of the Middle Yingshan Formation (O_1–2_ys) overlaid by the Lianglitag Formation (O_3_l) in the late O_1_-early O_3_. Tazhong area was entirely uplifted without large angular unconformity and experienced planation erosion. The palaeogeomorphology was relatively flat, without obvious karst plateau and karst slopes, indicating weak karst development (Fig. 6). Drilling data only showed karst voids filled by calcite, but no large caves. The thickness of karst development layers was relatively thin.

### High steep hill type karstification

This type was mostly located along the anticline thrust fold belt formed in the late Ordovician and Middle Devonian in the south Tarim Basin. Karstification occurred in the outcropped Ordovician carbonate rocks. Karst plateau was distributed along the fault zones on the land surface, while karst slopes were not well developed. The level of karst development decreased from the top weathering zone, to the phreatic zone, and to the slow flow karst zone in vertical direction. Typical karst features included large cave, weathered fractures, and void-cave-fracture combinations. However, caves were much less and smaller comparing to the Tahe type. Karst features of this type included: (1) karsts occurred in a wide range of depth as a result of high mountain steep-slope faulting, for example, TZ38 well-logging showed karsts at the 3485.9–3487.6 m depth, while Z3 well showed karst at the 3837.47–4638.30-m depth for a total depth difference of about 1100 meters. (2) karstification occurred in multiple layers due to structural movements in the late Ordovician and middle Devonian periods. Karst features were observed in the (O_3l_) limestone, in the interbedded O_1–2ys_ dolomatic limestone and limy dolomite, and in the O_1p_ dolomite. (3) karstification was highly correlated to lithology. The O_3l_ limestone was characterized by weathered fractures and karst voids filled with mud and calcites. The upper portion of the late Ordovician carbonate rocks was dominated by large caves, whereas the lower portion of the layer had more solutional holes, although some large cave could be seen.

### Covered and semi-open type karstification

In contrast to the three types discussed earlier, karstification for this type occurred in the middle and late Ordovician carbonate rocks overlain by upper Ordovician mudstone. However, karstification was more due to water cycle percolation from precipitation through fractures in the upper layer. This system was still an open karst system. This type of karst was located near the south end of the sloping area from the Tabei rift. The carbonate rock layer was covered by the upper Ordovician–Silurian mudstone and sandstone. Deep water cycles were largely driven by the elevation difference from north to south (Lv et al. 2009; Zhang et al. 2012), and karstification was largely controlled by fracture zones (zone B in Fig. 5). Some major features of this type include: (1) Karst cave and voids were mostly related to fractures. For example, number and dimension of cave were greater in the NS direction along and near the fracture zone and much less away from the fracture zone. (2) Karsts were distributed at a large range of depth from 300-to-1000 meters. (3) Fractures and voids were filled by chemical deposits and not much of materials from land surface. (4) No clear vertical zonation of karst and no obvious vadose zone karst features were noted (Lv et al. 2009).

## Conclusion

Thick carbonate rocks were deposited in the Cambrian–Middle Ordovician periods in the Tarim Craton Basin. The regional compressional tectonic movement during the Middle Caledonian–Hercynian resulted in the formation of Tazhong and Tabei paleouplifts, which lead to the outcropping of the Ordovician carbonate rocks, and subsequently, erosion and karstification. The paleogeomorphology, paleohydrogeology, and cycles of karst development in the Tarim Basin were controlled by multiple tectonic movements. Fractures created by structural activities in the basin provided favorable and necessary conditions for karstification by increased permeability and water cycle. Multi-stage stratigraphic denudation shaped paleokarst geomorphology, and such paleogeomorphology subsequently affected the development of karsts. Based on karst geomorphology, degree of karst development, and the layering of karst layer with overlying strata, the paleokarst in the Tarim Basin was classified into four types, i.e., the Tahe type (large angular unconformity), the gentle hill type (low angular unconformity-parallel unconformity), high steep hill type (angular unconformity), and covered and semi-open type (continuous stratigraphic un-outcropping). Relatively, the Tahe type had the strongest karstification, gentle hill type had weak karstification because of its peneplane karst geomorphology, and karstification of high and steep hill type was medium strong, but only located along or near the thrust fold zone. Karstification for the covered and semi-open type was mostly on the fracture zones and was controlled by fractures and hydraulic gradient from recharge-to-discharge zones. Areas far from the fracture zone had the weakest karstification.
